# Characterisation of the Bacterial and Fungal Communities Associated with Different Lesion Sizes of Dark Spot Syndrome Occurring in the Coral *Stephanocoenia intersepta*

**DOI:** 10.1371/journal.pone.0062580

**Published:** 2013-04-22

**Authors:** Michael Sweet, Deborah Burn, Aldo Croquer, Peter Leary

**Affiliations:** 1 Molecular Health and Disease Laboratory, School of Biology, Newcastle University, Newcastle Upon Tyne, United Kingdom; 2 Departamento de Estudios Ambientales, Universidad Simón Bolívar, Caracas, Venezuela; Charité-University Medicine Berlin, Germany

## Abstract

The number and prevalence of coral diseases/syndromes are increasing worldwide. Dark Spot Syndrome (DSS) afflicts numerous coral species and is widespread throughout the Caribbean, yet there are no known causal agents. In this study we aimed to characterise the microbial communities (bacteria and fungi) associated with DSS lesions affecting the coral *Stephanocoenia intersepta* using nonculture molecular techniques. Bacterial diversity of healthy tissues (H), those in advance of the lesion interface (apparently healthy AH), and three sizes of disease lesions (small, medium, and large) varied significantly (ANOSIM R  = 0.052 p<0.001), apart from the medium and large lesions, which were similar in their community profile. Four bacteria fitted into the pattern expected from potential pathogens; namely absent from H, increasing in abundance within AH, and dominant in the lesions themselves. These included ribotypes related to *Corynebacterium* (KC190237), *Acinetobacter* (KC190251), *Parvularculaceae* (KC19027), and *Oscillatoria* (KC190271). Furthermore, two *Vibrio* species, a genus including many proposed coral pathogens, dominated the disease lesion and were absent from H and AH tissues, making them candidates as potential pathogens for DSS. In contrast, other members of bacteria from the same genus, such as *V. harveyii* were present throughout all sample types, supporting previous studies where potential coral pathogens exist in healthy tissues. Fungal diversity varied significantly as well, however the main difference between diseased and healthy tissues was the dominance of one ribotype, closely related to the plant pathogen, *Rhytisma acerinum,* a known causal agent of tar spot on tree leaves. As the corals’ symbiotic algae have been shown to turn to a darker pigmented state in DSS (giving rise to the syndromes name), the two most likely pathogens are *R. acerinum* and the bacterium *Oscillatoria*, which has been identified as the causal agent of the colouration in Black Band Disease, another widespread coral disease.

## Introduction

Dark Spots Syndrome (DSS) is one of the most wide-spread and prevalent coral diseases/syndromes reported across the entire Caribbean region [1]. It was first documented as a discolouration of healthy coral tissues on Colombian reefs in 1990 [2], and has now been shown to occur throughout all areas of the Caribbean [3], [4]. DSS is known to primarily affect three species of hermatypic coral: *Stephanocoenia intersepta*, *Siderastrea siderea* and *Montastraea annularis* [5], although visual signs are different among these species (see Weil 2004). [6] showed that within the Florida Keys 71.2% of corals surveyed showing signs of disease were infected by DSS, highlighting that it is one of the most commonly occurring diseases in the region. Furthermore, [7] suggested that the disease may in fact affect many other species of coral further highlighting the importance of this syndrome in coral ecology and population biology. Interestingly, [5] highlighted significant differences in the morphology of the circular spots associated with these different coral species, and suggested that DSS might actually encompass more than one disease showing similar signs [1], [8], [9].

As the name suggests the disease characteristically manifests as dark spots on the coral surface which occur in a range of colours including brown, purple and black [5], [10], [11]. These lesions become depressed and tissue loss often follows. The exposed skeleton subsequently gets colonised by algae and/or boring sponges [12]. Although the visual signs of DSS can be seen on most Caribbean reefs, the aetiology of DSS, like many other coral diseases, still remains unknown and only a few studies have attempted to identify a specific pathogen; nonetheless both fungi [13] and bacteria [9] have been implied as potential causal agents to date. Characteristically, the *Symbiodinium* found in DSS lesions are swollen and darker in pigment in comparison to their natural healthy state with changes observed in their mitotic indices [5]. This led [5] to suggest that DSS is a pathology that acts primarily on the symbionts and secondarily on the coral host. In contrast, [10] argued that DSS is not a true disease but a disruption of the corals *Symbiodinium* due to a particular stress response such as increases in temperature and/or an immune response of the coral caused by irritation from boring organisms such as the sponge *Cliona delitrix* or *Siphonodictyon coralliphagu.*

From the analysis of histological sections of healthy tissues and DSS lesions affecting *S. siderea,* [13] found a fungus morphologically similar to *Aspergillus sydowii*, a microorganism that has been linked to Aspergillosis in sea fans [14], [15]. [13] also suggested that a digestive enzyme is being utilized by the coral as a defensive response to endolithic cellular invasion. In the same study, they highlighted cellular degradation and skeletal irregularities with the disease lesion, as well as a low abundance of *Symbiodinium*, many of which were abnormal with disruption of organelles [13]. In contrast to these studies, a bacterium similar to *Vibrio carchariae,* a pathogen previously linked to White Band Disease [16], has also be found to be unique to diseased samples and suggested as a potential bacterial causal agent of this coral syndrome, however [9] only utilised culture dependant techniques to analyse the bacterial communities associated with this syndrome, a technique which has been shown to largely underestimate the true microbial assemblages [17], [18]. To date there has been no culture independent studies on the microbial communities associated with Dark Spot and a simultaneous comparison with healthy samples of the same coral species. Previous studies have also ignored specific features of DSS with regard to sampling and analysis, such as the size of the lesions. Corals with DSS infections have multiple lesions which progress approximately 3 mm per month [10]. Therefore, it may be argued that lesion size could be a good indicator of age of infection, and therefore provide information about temporal changes of microbial assemblages as the pathology progresses. As a first step towards understanding causation of the field signs associated with DSS, this study provides a comprehensive, culture independent molecular analysis of both bacterial and fungal communities associated with the disease on the dominant reef coral *Stephanocoenia intersepta*. Furthermore we analysed the microbial communities associated with three different sized disease lesions of Dark Spot (small lesions: 1–2 cm in diameter, medium lesions: 5–10 cm and large lesions: >10 cm) for which we are inferring as an indication to infection age.

## Materials and Methods

### Sample collection

**Figure 1:**
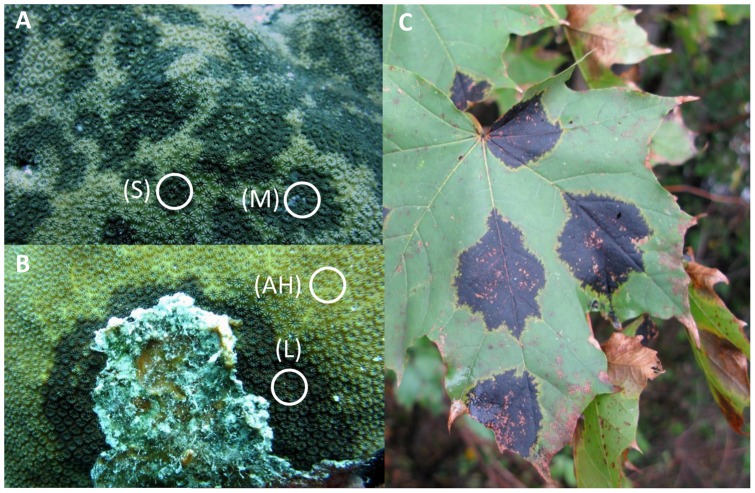
*Stephanocoenia intersepta* showing Dark Spots Syndrome. (a) shows a heavily infested colony with variable sizes of dark spots, (S) is classed as a small spot and (M) classed as a medium spot, (b) shows the characteristic dead tissue which follows this syndrome usually associated with larger spots (L), (AH)  =  Apparently Healthy tissues sampled for direct comparisons to the diseased lesions, and (c) is a reference shot of the terrestrial plant disease known as Tar Spot caused by the fungal pathogen *Rhytisma acernium*, the same pathogen of which was isolated from DSS lesions and absent in healthy tissues.

The samples consisted of 5 cm^2^ tissues comprising of healthy tissue from a coral showing no signs of DSS (H; n = 5), apparently healthy tissue >5 cm from the lesion interface on a coral showing signs of DSS (AH; n = 5), and the disease lesion itself for small (S-DL; n = 5), medium (M-DL; n = 5) and large (L-DL; n = 5) spot sizes (Fig. 1). These samples were collected using a sterile hammer and chisel from randomly selected colonies of *Stephanocoenia intersepta* at a depth range of 13–17 m. The samples were collected with permission of the Instituto Nacional de Parques (INPAQUES) of Venezuela. Surgical gloves were worn to avoid contamination, and the samples were placed in falcon tubes which were sealed before being transferred to the surface and stored on ice before preservation in the laboratory. All samples were preserved in 100% ethanol for DNA extraction and stored at −20°C until extraction.

### PCR, DGGE and Clone Libraries

Extraction, PCR and denaturing gradient gel electrophoresis were undertaken as described in [19]. DNA was extracted from all samples using QIAGEN DNeasy Blood and Tissue kits with an added step to concentrate the lysate using a vacuum centrifuge for 4 hours at 24°C. Bacterial 16S rRNA gene diversity was amplified using standard prokaryotic primers; (GC-357F) (5′- CGCCCGCCGCGCGCGGCGGGCGGGGCGGGGGCAGCACGGGGGGCCTACGGGAGGCAGCAG-3′) and (518R) (5′-ATTACCGCGGCTGCTGG-3′). Thirty PCR cycles were performed at 94°C for 30 sec, 53°C for 30 sec and 72°C for 1 min, and a final extension at 72°C for 10 min [20]. For fungi, a nested PCR approach was utilised as this allows greater specificity with regard to the fungal primers. Primers targeting the ITS region of fungi were used; ITS1F (5′-CTTGGTCATTTAGAGGAAGTAA-3′) and ITS4F (5′-TCCTCCGCTTATTGATATGC-3′) were run initially following the protocol described by [21] (at 94°C for 5 min; followed by 35 cycles at; 94°C for 30 sec, 55°C for 30 sec, 72°C for 30 sec then elongation at 72°C for 5 min). Then a 1∶100 dilution of the PCR product was then used in a further PCR with primers ITS3F (5′-GCATCGATGAAGAACGCAGC-3′) and ITS4F-GC (5′CGCCCGCCGCGCCCCGCGCCCGGCCCGCCG CCCCCGCCCCTCCTCCGCTTATTGATATGC-3′) [22]. For all of the above reactions, 30 µl PCR mixtures containing 1.5 mM MgCl_2_, 0.2 mM dNTP (PROMEGA), 0.5 µM of each primer, 2.5 Ul of Taq DNA polymerase (QBiogene), incubation buffer and 20 ng of template DNA [23] were carried out on a Hybaid PCR Express thermo cycler. PCR products were verified by agarose gel electrophoresis (1.6% (w/v) agarose) with ethidium bromide staining and visualized using a UV transilluminator.

DGGE was performed using the D-Code universal mutation detection system (Bio-Rad). PCR products were resolved on a 10% (w/v) polyacrylamide gels that contained a 30–60% denaturant gradient for 13 hours at 60°C with a constant voltage of 50 V. Gels were stained as per [24] and visualized using a UV transilluminator. Bands of interest (those which explained the greatest differences/similarities between samples) were excised from DGGE gels, left overnight in Sigma molecular grade water, vacuum-centrifuged, re-amplified with the same original primers, labelled using Big Dye transformation sequence kit (Applied Biosystems) and sent to Genevision (Newcastle University, UK) for sequencing. Operational taxonomic units (OTUs) were defined from DGGE band-matching analysis using Bionumerics 3.5 (Applied Maths BVBA). Standard internal marker lanes were used to allow for gel to gel comparisons. Tolerance and optimization for band matching was set at 1%. PCR.

### Clone Libraries and ARDRA screening

Almost-complete 16S rRNA gene fragments were amplified from the DNA extracted using the ‘universal’ eubacterial 16S rRNA gene primers 27F, (5′-AGAGTTTGATCGTGGCTC AG-3′) and 1542R, (5′-AAGGAGGTGATCCAGCCGCA-3′) [25], [26]. Ten PCR cycles were performed at 94°C for 1 min, 55°C for 1 min and 72°C for 3 min followed by a further twenty five cycles at 94°C for 1 min, 53°C for 1 min and 72°C for 3 min with a final extension at 72°C for 10 min. The amplified products were purified using the Qiagen PCR purification kit, inserted into the pGEM-T vector system (Promega) and transformed into *Escherichia coli* JM109 cells. A total of 392 clones containing the 16S rRNA gene inserts were randomly selected from each sample/replicate, and boiled lysates were prepared from each by mixing a picked clone in 30 μl of TE and boiled for 3 min followed by freezing. Each lysate (1 µl) was amplified using the primers pUCF (5′ -CTA AAA CGA CGG CCA GT- 3′) and pUCR (5′ -CAG GAA ACA GCT ATG AC- 3′). Twenty five PCR cycles were performed at 94°C for 1 min, 55°C for 1 min and 72°C for 1 min with a final extension at 72°C for 10 min. The products were then digested with the restriction enzymes HaeIII and RsaI (Promega). 4 µg of PCR product was used with 2 µl of restriction buffer (10 mM Tris at pH 7.6, 10 mM NaCl and 10 mM MgCl_2_), 0.002mg of Bovine Serum Albumin (BSA) and 1 unit/µg DNA of each of the two restriction enzymes (which equates to 0.07 µl of HaeIII and 0.1 µl of RsaI). The final volume was made up to 20 µl with sigma water and digested for 2 h at 37°C then 10 min at 67°C. Restriction fragments were resolved by electrophoresis on a 3% agarose gel, visualized using a UV transilluminator and grouped based on restriction patterns. Representatives from each group were sequenced. Closest match of retrieved sequences was determined by RDP II similarity matching [27]. All sequences in this study have been deposited in GenBank and their unique accession numbers reported in the text (KC190223-KC190272).

### Statistical Analysis

An analysis of similarity (ANOSIM) was conducted to test differences in 16S rRNA gene bacterial assemblage and ITS rRNA gene fungal assemblages between samples (H, AH, S-DL, M-DL and L-DL). Percentage similarity (SIMPER) was performed to determine which ribotypes better explained differences and/or similarities between sample types. Patterns of the 16S rRNA gene bacterial assemblages were represented on a multidimensional scaling (MDS) plot.

## Results

### Description of bacterial assemblages

**Figure 2:**
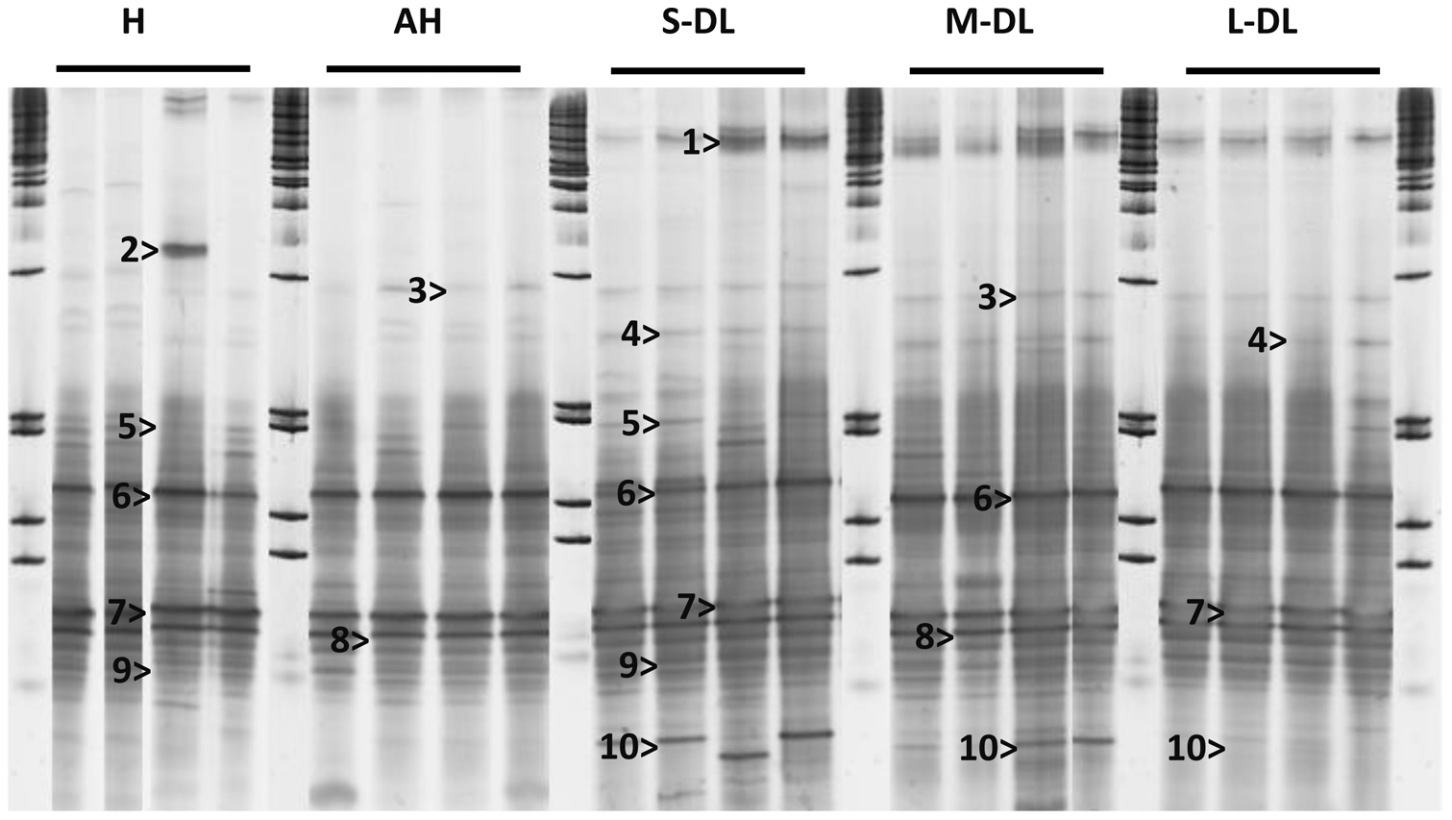
A Denaturalized Gradient Gel Electrophoresis (DGGE) image, showing sequenced bands (16S rRNA gene bacterial ribotypes) which gave the greatest similarities or differences between sample types. Healthy (H), Apparently Healthy (AH), Small Disease Lesion (S-DL), Medium Disease Lesion (M-DL) and Large Disease lesions (L-DL) from corals showing signs of Dark Spot Syndrome. Marker lanes between sample types allow for gel to gel comparisons when analysed using BioNumerics. >1 =  *Vibro* sp. (JX407129), >2 =  *Acidovorax* sp. (JQ595481), >3 =  *Caulobacter* sp. (FJ581041), >4 =  *Vibrio harveyi* (EU716714), >5 =  *Pseudomonas* sp (HQ706105), >6 =  *Sphingomonas* sp. (HM223578), >7 =  *Cyanobacterium* sp. (GU590842), >8 =  *Acinetobacter* sp. (JX391982), >9 =  *Campylobacter* sp. (NR_042684) and >10 =  *Photobacterium* sp. (JX407239).

**Figure 3:**
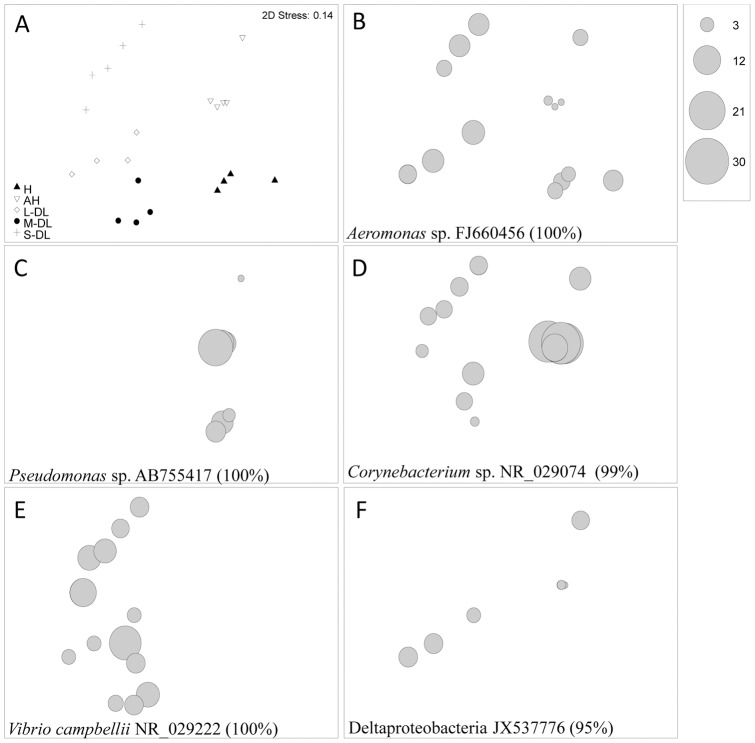
Non Metric Multidimensional scaling (MDS) plots. (a) shows changes in 16S rRNA gene bacterial communities between healthy (H), apparently healthy (AH) and three different sized disease lesions; Small Disease Lesion (S-DL), Medium Disease Lesion (M-DL) and Large Disease lesions (L-DL) from corals showing signs of Dark Spot Syndrome. (b-f) MDS bubble plots of a subset of bacterial ribotypes derived from SIMPER analysis which gives the ribotypes offering the greatest differences or similarities between sample types.

**Figure 4:**
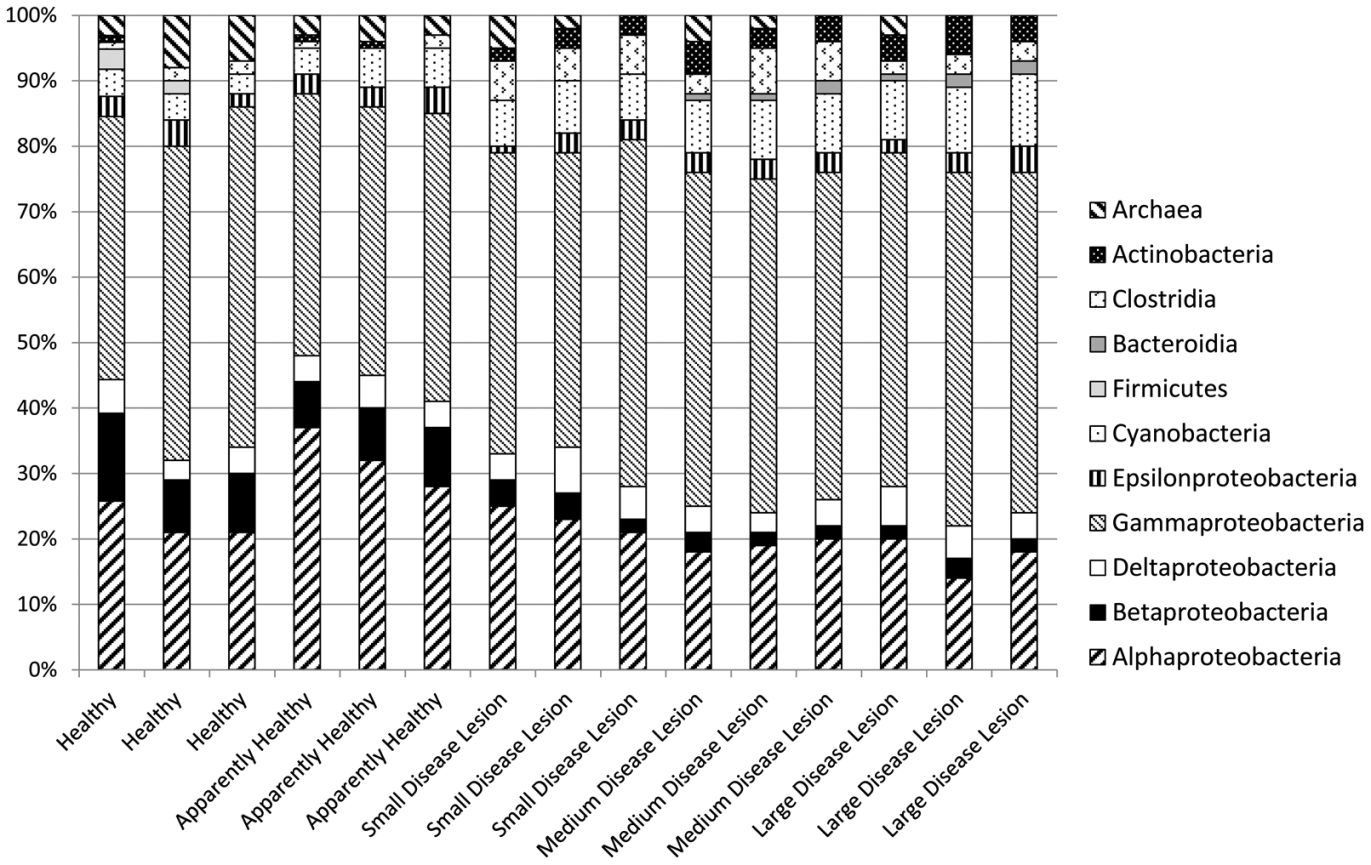
Bar chart illustrating 16S rRNA gene bacterial clone libraries separated into class from replicate samples of Healthy, Apparently Healthy, and Diseased *Stephanocoenia intersepta* showing signs of Dark Spot Syndrome.

There was a significant difference in 16S rRNA gene bacterial diversity between all sample types, H, AH, S-DL, M-DL and L-DL in both the DGGE (Fig. 2, 3) and Clone Libraries (Fig. 4, Table 1, ANOSIM R = 0.921 p<0.001 and ANOVA R = 0.952 p<0.001 respectively). Pairwise comparisons showed that microbial assemblages present in all sample types were different, the only exception being the ones associated with the M-DL and the L-DL (Pairwise ANOSIM R = 0.521 p = 0.057). Bacteria such as a *Pseudoxanthomonas* sp. (KC190223), *Aeromonas* sp. (KC190224), *Desulfacinum* sp. (KC190239), *Campylobacter* sp. (KC190240), *Pseudomonas* sp. (KC190242), *Clostridium* sp. (KC190233) and an *Enterobacter* sp. (KC190227) were present throughout all sample types including all disease lesion-sizes (Table 1). Two *Vibrio* species (GenBank Accession Nos. KC190244 & KC190245) were also detected in all sample types, one of which had a 100% match in the BLAST search to the proposed coral pathogen *V. harveyi* (EU716714). Some ribotypes were only present in H and AH samples and absent from all disease lesion sizes. These included ribotypes similar to a *Burkholderia* sp. (KC190268), a *Pseudomonas* sp. (KC190241), a *Parvibaculum* sp. (KC190259) and an *Ochrobactrum* sp. (KC190269) (Table 1). In contrast, three ribotypes; a *Photobacterium* sp. (KC190243) and two further *Vibrio* species, one of which was a 100% BLAST match to *V. campbellii* (NR_029222) dominated all three disease lesion sizes and were absent from H and AH tissues (Table 1). Only four bacteria were absent from H, increased in AH and were dominant in all the diseased tissues, these included ribotypes related to a *Corynebacterium* sp. (KC190237), an *Acinetobacter* sp. (KC190251), a *Parvularculaceae* sp. (KC190270) and an *Oscillatoria* sp. (KC190271).

**Table 1:** Heatmap showing replicate 16S rRNA gene clone libraries of Healthy (H), Apparently Healthy (AH), Small Disease Lesion (S-DL), Medium Disease Lesion (M-DL) and Large Disease lesions (L-DL) from corals showing signs of Dark Spot Syndrome.

GenBank Accession No.	Genus	Closest Relative	% Match	Class	H	H	H	AH	AH	AH	S-DL	S-DL	S-DL	M-DL	M-DL	M-DL	L-DL	L-DL	L-DL
KC190223	*Pseudoxanthomonas*	JX576006	99%	Gammaproteobacteria	3	5	6	4	2	5	4	2	5	4	5	5	5	4	6
KC190224	*Aeromonas*	FJ660456	100%	Gammaproteobacteria	5	4	5	2	2	3	3	4	3	4	2	3	3	3	4
KC190225	*Vibro*	JX407129	100%	Gammaproteobacteria	0	0	0	0	0	0	2	2	3	3	3	2	4	5	2
KC190226	*Alteromonas*	HQ439533	99%	Gammaproteobacteria	3	5	5	2	5	4	3	5	3	2	6	5	4	4	5
NA	*Methanococcus*	NR_029140	95%	Archaea	2	4	3	1	0	0	0	1	0	3	2	0	1	0	0
KC190230	*Corynebacterium*	FJ423600	100%	Actinobacteria	4	0	0	0	0	0	0	0	0	1	1	1	1	3	2
KC190227	*Enterobacter*	JF772064	99%	Gammaproteobacteria	2	5	2	2	4	3	4	4	5	4	5	4	4	3	4
KC190228	*Klebsiella*	EF576996	100%	Gammaproteobacteria	2	3	4	4	3	2	4	5	3	4	4	4	3	4	3
KC190229	*Burkholderia*	HM223571	99%	Betaproteobacteria	4	1	3	2	2	0	0	0	0	0	0	0	0	0	0
KC190241	*Pseudomonas*	AB755417	100%	Gammaproteobacteria	5	4	6	4	3	3	0	0	0	0	0	0	0	0	0
KC190231	*Sulfurisphaera*	NR_043432	100%	Archaea	1	4	4	2	4	3	4	1	0	0	0	0	0	0	0
KC190232	*Balneola vulgaris*	NR_042991	100%	Bacteroidia	0	0	0	0	0	0	0	0	0	1	1	2	1	2	2
KC190233	*Clostridium*	NR_028611	94%	Clostridia	1	2	2	1	0	2	4	2	4	3	4	3	2	3	3
KC190242	*Pseudomonas*	JQ734667	98%	Gammaproteobacteria	2	1	4	1	2	2	4	1	4	4	4	4	3	4	3
KC190234	*Methanococcus*	NR_028861	95%	Archaea	0	0	0	0	0	0	1	0	0	1	0	0	2	0	0
KC190243	*Photobacterium*	JX407239	100%	Gammaproteobacteria	0	0	0	0	0	0	1	2	4	3	3	3	2	3	3
KC190235	*Clostridium*	NR_029271	100%	Clostridia	0	0	0	0	0	0	2	3	2	0	3	3	0	0	0
KC190236	*Vibrio campbellii*	NR_029222	100%	Gammaproteobacteria	0	0	0	0	0	0	3	4	3	3	3	3	3	4	4
KC190237	*Corynebacterium*	NR_029074	99%	Actinobacteria	0	0	0	1	1	0	2	3	3	4	2	3	3	3	2
KC190238	*Rhodobacter*	NR_029215	100%	Alphaproteobacteria	3	6	4	1	5	5	2	2	0	0	0	0	1	0	0
KC190239	*Desulfacinum*	NR_028757	95%	Deltaproteobacteria	5	3	4	1	3	3	4	4	3	4	3	4	5	3	3
KC190240	*Campylobacter*	NR_042684	94%	Epsilonproteobacteria	3	4	2	3	3	4	1	3	3	3	3	3	2	3	4
KC190244	*Vibrio harveyi*	EU716714	100%	Gammaproteobacteria	2	2	2	2	2	3	4	3	3	3	2	3	3	3	3
KC190245	*Vibrio proteolyticus*	HQ340600	100%	Gammaproteobacteria	4	6	4	3	3	3	4	2	4	4	3	3	3	4	4
KC190246	*Sphingobium*	JN700069	99%	Alphaproteobacteria	0	0	0	3	1	2	2	3	3	0	0	0	0	0	0
KC190247	*Unknown*	JQ580172	98%	Deltaproteobacteria	0	0	0	2	0	0	0	3	2	0	0	0	0	0	0
KC190248	*Acinetobacter*	JX391982	98%	Gammaproteobacteria	2	4	2	2	3	3	2	4	3	4	4	3	3	3	4
KC190249	*Chromatiaceae*	HQ003530	95%	Gammaproteobacteria	0	2	3	2	1	1	0	0	1	2	0	0	3	3	1
KC190250	*Acidovorax*	JX390645	99%	Betaproteobacteria	4	3	1	0	2	2	3	2	2	3	2	2	2	3	2
KC190251	*Acinetobacter*	JX486707	98%	Gammaproteobacteria	0	0	0	2	1	2	1	3	4	3	4	4	4	3	3
KC190252	*Unknown*	FJ425615	95%	Gammaproteobacteria	0	0	0	0	0	1	0	0	0	0	0	0	2	0	0
KC190253	*Unknown*	GQ349065	96%	Firmicutes	3	2	0	0	0	0	0	0	0	0	0	0	0	0	0
KC190254	*Unknown*	AB294937	95%	Alphaproteobacteria	0	0	0	3	1	1	1	2	0	0	0	0	0	0	0
KC190255	*Cyanobacterium*	GU590842	95%	Cyanobacterium	3	4	3	2	3	4	4	4	4	4	4	4	3	4	4
KC190256	*Caulobacter*	FJ581041	98%	Alphaproteobacteria	6	3	3	5	5	4	5	5	5	5	4	4	3	4	6
KC190257	*Shewanella*	GU289647	100%	Gammaproteobacteria	1	1	0	0	2	3	3	0	1	4	3	4	2	3	2
KC190258	*Acidovorax*	JQ595481	97%	Betaproteobacteria	0	0	1	0	0	1	1	0	0	0	0	0	0	0	0
KC190259	*Parvibaculum*	FJ356664	95%	Alphaproteobacteria	3	2	1	5	3	2	0	0	0	0	0	0	0	0	0
KC190260	*Acinetobacter*	EU221346	98%	Gammaproteobacteria	5	4	5	6	5	4	0	0	0	0	0	0	0	0	0
KC190261	*Unknown*	GU319260	95%	Alphaproteobacteria	0	0	0	0	0	1	2	0	1	2	0	2	3	0	0
KC190262	*Unknown*	AY654810	94%	Alphaproteobacteria	0	0	0	0	1	0	0	0	2	1	1	0	0	0	0
KC190263	*Sphingomonas*	HM223578	100%	Alphaproteobacteria	4	3	4	4	5	3	3	4	5	4	5	5	5	4	5
KC190264	*Unknown*	HM223576	95%	Alphaproteobacteria	0	0	0	0	1	1	2	2	0	0	0	1	2	1	2
KC190265	*Unknown*	HM223568	95%	Gammaproteobacteria	0	0	0	0	0	0	0	0	0	0	0	0	0	1	1
NA	*Unknown*	HM223562	95%	Deltaproteobacteria	0	0	0	0	0	0	0	0	0	0	0	0	1	2	1
KC190266	*Unknown*	HM223544	94%	Alphaproteobacteria	1	1	3	4	1	1	0	0	0	0	0	0	0	0	0
KC190267	*Sphingomonas*	JF835729	99%	Alphaproteobacteria	5	3	2	5	4	4	5	2	1	1	3	3	1	1	2
KC190268	*Burkholderia*	JN975200	97%	Betaproteobacteria	5	4	5	5	4	6	0	2	0	0	0	0	0	0	0
KC190269	*Ochrobactrum*	HM223545	98%	Alphaproteobacteria	3	3	3	6	4	3	0	0	0	0	0	0	0	0	0
NA	*Unknown*	JX537776	95%	Deltaproteobacteria	0	0	0	1	2	1	0	0	0	0	0	0	1	0	1
KC190270	*Parvularculaceae*	FJ516787	98%	Alphaproteobacteria	0	0	0	1	1	1	3	3	4	5	6	5	5	4	3
KC190271	*Oscillatoria*	DQ917838	99%	Cyanobacteria	0	0	0	2	3	2	3	4	3	4	5	5	5	6	6
KC190272	*Pseudomonas*	HQ706105	100%	Gammaproteobacteria	4	2	4	4	3	2	4	4	4	0	0	0	0	0	0

The M-DL and L-DL shared similar bacterial communities (with an average dissimilarity of 47%), whilst a significant difference in the bacterial communities of the S-DL occurred between both the M-DL and the L-DL (average dissimilarity 61% & 53% respectively). The main differences seen between disease lesions were driven by a Delta-proteobacteria (KC190247) that was dominant in S-DL and absent in all other sample types. An alpha-proteobacteria (KC190254) and a *Sphingobium* sp. (KC190246) were present in the S-DL and the AH tissues but absent in other types and a *Pseudomonas* sp. (KC190272) was present in H, AH and the S-DL but absent in both the M-DL and L-DL. Only one bacterial ribotype, a *Corynebacterium* sp. (KC190230) was present in M-DL and L-DL yet absent in the S-DL. Average Bray-Curtis similarities for all tissues sampled (H, AH, TR, S-DL, M-DL and L-DL) were above 80%, further indicating low variability in bacterial assemblages among replicates (Table 2). In each of these samples, diverse bacterial assemblages explained up to 80% of the high BC-similarities observed (Table 2).

**Table 2:** Relative contribution (%) of bacterial 16S rRNA gene ribotypes sequenced from clone libraries, indicating the average contribution of each bacteria to the similarity within each sample type based on SIMPER analysis.

	Average similarity	85.66	82.81	82.11	89.81	88.17
GenBank Accession No.	ID	H	AH	S-DL	M-DL	L-DL
KC190223	*Pseudoxanthomonas*	4.11	3.31	3.46	4.27	4.21
KC190224	*Aeromonas*	4.5	2.91	3.72	3.12	3.51
KC190225	*Vibro*	­	­	3.04	3.12	3.26
KC190226	*Alteromonas*	4.11	3.3	3.72	3.47	4.05
NA	*Methanococcus*	3.29	­	­	­	­
KC190230	*Corynebacterium*	­	­	­	­	2.31
KC190227	*Enterobacter*	3.06	3.12	4.3	4.11	3.69
KC190228	*Klebsiella*	3.29	3.13	3.91	4.11	3.51
KC190229	*Burkholderia*	2.69	­	­	­	­
KC190241	*Pseudomonas*	4.5	3.56	­	­	­
KC190231	*Sulfurisphaera*	2.89	3.12	­	­	­
KC190232	*Balneola vulgaris*	­	­	­	­	2.31
KC190233	*Clostridium*	­	­	3.46	3.56	3.08
KC190242	*Pseudomonas*	2.46	2.34	2.87	4.11	3.51
KC190243	*Photobacterium*	­	­	2.445	3.56	3.08
KC190235	*Clostridium*	­	­	3.04	­	­
KC190236	*Vibrio campbellii*	­	­	3.72	3.56	3.69
KC190237	*Corynebacterium*	­	­	3.27	3.12	3.08
KC190238	*Rhodobacter sp.*	3.94	2.9	­	­	­
KC190239	*Desulfacinum*	3.94	2.55	3.91	3.74	3.51
KC190240	*Campylobacter*	3.29	3.56	2.68	3.56	3.08
KC190244	*Vibrio harveyi*	3.06	2.91	3.72	3.12	3.51
KC190245	*Vibrio proteolyticus*	4.33	3.56	3.46	3.56	3.69
KC190246	*Sphingobium*	­	2.34	3.27	­	­
KC190248	*Acinetobacter*	3.06	3.12	3.27	3.74	3.51
KC190249	*Chromatiaceae*	­	2.06	­	­	2.51
KC190250	*Acidovorax*	2.69	­	3.04	2.9	2.86
KC190251	*Acinetobacter*	­	2.34	2.68	3.74	3.51
KC190254	*Unknown*	­	2.06	­	­	­
KC190255	*Cyanobacterium*	3.75	3.12	4.3	4.11	3.69
KC190256	*Caulobacter*	3.75	4.27	4.8	4.11	3.69
KC190257	*Shewanella*	­	­	­	3.74	2.86
KC190259	*Parvibaculum*	­	3.13	­	­	­
KC190260	*Acinetobacter*	4.5	4.27	­	­	­
KC190263	*Sphingomonas*	3.94	3.75	3.91	4.27	4.21
KC190266	*Unknown*	­	2.06	­	­	­
KC190267	*Sphingomonas*	3.29	4.11	­	2.56	­
KC190268	*Burkholderia*	4.5	4.27	­	­	­
KC190269	*Ochrobactrum*	3.75	3.75	­	­	­
KC190270	*Parvularculaceae*	­	­	3.72	4.59	3.69
KC190271	*Oscillatoria*	­	2.91	3.72	4.27	4.68
KC190272	*Pseudomonas*	3.48	3.13	4.3	­	­

Healthy (H), Apparently Healthy (AH), Small Disease Lesion (S-DL), Medium Disease Lesion (M-DL) and Large Disease lesions (L-DL).

### Description of fungal assemblages

There was a significant difference in ITS fungal diversity between H, AH and all sizes of the diseased lesions (ANOSIM, R = 0.897 p = 0.01), but not between the different sizes of lesion (ANOSIM, R = 0.928 p = 0.89). There was a much lower diversity of fungi detected in all sample types when compared to the number of bacterial ribotypes in the same samples. Only four dominant ribotypes were present in all samples; these included a *Tritirachium* sp. GenBank Accession No. KC521539 (100% match to JF779666), a *Cryptococcus* GenBank Accession No. KC521540 (100% match to AM748529), a *Malasseziales* sp. GenBank Accession No. KC521541 (100% match to EU812484) and *Aspergillus sydowii* GenBank Accession No. KC521542 (100% match to JQ647895). Only one ribotype (KC521543) was found consistently in diseased samples and absent in all other sample types. This ribotype returned a 100% BLAST match to a novel strain of the terrestrial plant pathogen *Rhytisma acerinum* (GQ253100).

## Discussion

Over the past few decades, there has been controversy over whether DSS is a disease or simply an immune response of the coral to a particular stress. While some studies find no evidence of tissue mortality associated with this syndrome [28], others have shown DSS lesions to progress at a rate of approximately 3–4 cm month^−1^ [5], [10]. Despite this controversy over potential tissue loss caused by DSS, all authors agree that DSS is persistent when it is detected and compromises the overall health of the coral. Time series images taken of corals showing signs of DSS by [10] showed that when tissue loss occurs large colonies fragment to smaller ramets. Moreover, tissues associated with DSS lesions have never been shown to recover; and areas of exposed skeleton are usually colonised by algae or boring sponges [5]. Therefore with this in mind we refer to DSS as a disease in the rest of the discussion.

### Difference in bacterial and fungal communities between healthy and diseased tissues

There was a significant shift in the 16S rRNA gene bacterial diversity between healthy and diseased tissues. This shift was also noticed to occur in advance of the disease lesion interface within apparently healthy tissues, a result seen by an increasing number of studies [19], [29], [30]. Temporal changes of bacterial communities throughout coral disease progression are poorly understood as descriptions of these communities in most of the studies do not provide information about age of infection. In this study, there are significant differences in the dominant bacterial diversity between smaller lesions and those associated with the medium/large lesions; this difference may be due to the age of infection, a result previously seen in other coral diseases such as Black Band Disease (BBD) [31]. Using culture-independent techniques, [31] found shifts within cyanobacterial assemblages present in BBD samples. Bacteria such as *Blennothrix* sp. were found to dominate early BBD lesions, whilst *Oscillatoria* sp. was found to dominate older lesions [32]. In general the corals were dominated by Alphaproteobacteria in early stages of BBD which subsequently shifted to a community dominated by Gammaproteobacteria and Cyanobacteria in the latter stages of disease. Similarly, in this study we saw a reduction in Alphaproteobacteria dominance and an increase in Cyanobacteria and Gammaproteobacteria. Interestingly, we also saw an increase in the number of Actinobacteria in the larger disease lesions compared to both the healthy tissues and smaller lesion sizes.

*S. intersepta* was shown to contain potentially pathogenic bacteria and fungi such as *Vibrio harveyi and Aspergillus sidowii*, in both healthy as well as diseased tissues, following patterns observed by numerous studies [19], [29], [30], [33]. [9] used culture dependent molecular techniques and showed that a bacterium closely related to *V. carchariae* was associated with three species of coral showing signs of DSS (*Montastraea annularis, M. faveolata* and S*iderastrea siderea*) and they reported that *V. carchariae* was not detected within healthy tissues of the same corals. However, inoculation experiments with this bacterium failed to reproduce the classical signs of the disease, suggesting that *V. carchariae* is not a causal agent of DSS in these corals and more likely a coloniser of the dead or dying tissues or the exposed bare calcium carbonate skeleton [19]. In this study, we employed two separate culture independent molecular screening techniques, which utilised two different sets of universal bacterial primers allowing us to better describe the 16S rRNA gene bacterial diversity of healthy and DSS affected corals. Although no BLAST hits matched any sequence related to *V. carchariae* in our samples, there were two other ribotypes related to two different *Vibrio* sp. These *Vibrios*, similar to the result found by [9] were present only in diseased tissues and absent in healthy samples, one of which was a 100% match to *V. campbellii.* However, it is likely that these two bacteria play similar roles to that of *V. carchariae,* and are not directly involved in the disease itself. Although further experiments would need to be conducted to confirm this such as inoculation tests to determine whether these bacteria are pathogenic to the corals. Fungal diversity was much lower than that observed for bacteria in all samples, with only five dominant ribotypes detected. Four of these were detected in all samples and show that fungi play a role in the natural coral holobiont. However, in contrast one specific ribotype (KC521543) was detected solely in diseased tissues, which was identical (100% BLAST match) to a newly identified strain of the plant pathogen known to cause tar spot disease in tree leaves (Fig. 1c), *Rhytisma acernium* [34], [35].

### Potential pathogens associated with diseased lesions

Previous studies have shown that DSS causes cellular degradation, vacuolization and necrosis in affected tissues [13]. The presence of endolithc fungi has previously been shown in DSS in *S. siderea*, yet absent in healthy samples. These were accredited at the time to being morphologically as similar to *Aspergillosis sydowii*, the fungal pathogen thought to cause the purple discolouration and necrotic tissue loss in gorgonian sea fans [36]–[38]. In this study, although we looked at a different coral species *Stephanocoenia intersepta*, we show that although *A. sydowii* was detected in the samples, it was found in the same abundance in all tissue types both healthy and diseased, a result similar to that found recently within *Gorgonia sp.* [39]. In contrast, *R. acernium* (KC521543), was identified in this study to be present only in diseased corals showing signs of DSS and not in the healthy tissues. *R. acernium* in the terrestrial environment affects the chloroplasts of the leaves leaving them in a darkened pigmented state (*re* the name tar spot). As the corals *Symbiodinium* have been shown to be directly affected by DSS in other coral species, *R. acernium* (KC521543) may be having a similar effect on the corals symbiotic algae as it does on terrestrial plants chloroplasts. However this needs further investigation.

With regard to bacteria, we showed an increase in two main classes (*Cyanobacteria* and *Actinobacteria*) within all disease lesion sizes. The Cyanobacteria were dominated by an *Oscillatoria* sp., the same genus of which has been implicated as a causal factor of another wide-spread coral disease, Black Band Disease, [25]. *Oscillatoria* have been shown to cause the dark black band associated with BBD, however in this study although in lower abundance we did detect the bacterium in one sample of the healthy tissues. Therefore, although *Oscillatoria* may contribute to the dark pigmentation it is unlikely to be a specific bacterial pathogen causing DSS. Similarly, *Actinobateria* were found to be significantly more abundant within the lesions in this study, yet have been previously shown to be present in healthy and diseased corals in other studies [40]–[42]. Interestingly, many *Actionbacteria* have been shown in terrestrial environments to be capable of degrading plant tissues [43] and therefore might have some role in the degradation of the symbiotic algae in DSS, although their presence in healthy corals suggests this is unlikely. Furthermore, *Actinobacteria* studied in other systems have been shown to have significant anti-fungal capabilities [44], [45], therefore an alternate hypothesis to this increase in *Actinobacteria* in diseased lesions might be that they are acting as a defensive response for the coral, potentially fighting off the fungal pathogen, *R. acernium* (KC521543).

In conclusion, we highlight numerous potential pathogens likely involved in the disease in some respect including some bacterial pathogens (*Corynebacterium* sp., *Acinetobacter* sp., *Parvularculaceae* sp., *Oscillatoria* sp. and two *Vibrio* sp.) and one fungal ribotype (*Rhytisma acernium* – KC521543). Similarly to that shown previously for BBD, we show that bacterial communities vary with size of lesion and is therefore likely related to age of infection. Interestingly, [46] suggest that White Plague (WP) and DSS might be the same disease, representing even further stages of progression than studied here. If this is the case monitoring of colonies infected with small lesions over time and sampling continuously will allow for further microbial analysis to be conducted addressing any further shifts in the microbial communities associated with DSS and potentially WP. Regardless of if these diseases are the same or not, the results from this study, in combination with evidence from previous studies on this disease, has led us to hypothesis that a novel marine strain of the fungal pathogen, *R. acernium* (KC521543), is a more likely causal agent of DSS than the potential bacterial pathogens detected in this study. The similarities of DSS to the terrestrial plant disease tar spot are striking and warrant immediate further studies aimed to fulfil Kochs postulates.
